# Genetic analysis of rapidly progressing esophageal squamous cell carcinoma: A case report

**DOI:** 10.1097/MD.0000000000024462

**Published:** 2021-03-05

**Authors:** Shuang Zhao, Ni Sun, Xi Yuan, Zetian Shen, Xixu Zhu, Jing Li

**Affiliations:** Department of Radiation Oncology, Jinling Hospital, Medical School of Nanjing University, Jiangsu, China.

**Keywords:** esophageal squamous cell carcinoma, genetic analysis, *PIK3CA*, TP53

## Abstract

**Introduction:**

Numerous investigations have been performed to explore candidate biomarker proteins in esophageal squamous cell carcinoma (ESCC) patients, which could predict the response to chemoradiotherapy (CRT). Here we report a patient with unresectable ESCC who had unsatisfactory effects with radiotherapy, chemotherapy and immunotherapy. We performed genetic analysis in this patient to gain insights about the cause of the rapid progression.

**Patient concerns:**

A 65-year-old man presented with food obstruction, hoarse voice and choking on drinking water for 2 months, and pain behind the breastbone for 1 month.

**Diagnosis:**

The patient was clinically diagnosed with ESCC and staged as T4N1M1 Stage IV

**Interventions:**

The patient was treated with CRT and immunotherapy. Mutational analyses through high throughput DNA sequencing methodology (next generation sequencing; NGS) was performed on the patient's blood sample.

**Outcomes:**

The tumor progressed rapidly during the treatment period, and the patient passed away only 3 months from the onset of symptoms.

**Conclusion:**

Although the role of TP53 gene and *PIK3CA* gene in the progression, treatment and sensitivity of esophageal cancer has been studied, the mechanism of their simultaneous appearance has not been demonstrated in relevant studies. We speculate that the reason for the rapid progression in this patient during active treatment might be related to this. Further studies are needed to validate our observations.

## 1 Introduction

Esophageal cancer (EC) is one of the most common and aggressive tumors, which is a leading cause of cancer-related deaths worldwide.^[1,2]^ Esophageal squamous cell carcinoma (ESCC) is the most common pathological type of esophageal cancer, and the complex carcinogenic mechanism of ESCC is related to poor prognosis in China.^[1,3,4]^ Since the NCCN guidelines strongly recommend definitive chemoradiotherapy (CRT) for unresectable ESCC, the efficacy of CRT varies greatly among different patients.^[5]^ Numerous researches have explored the candidate biomarker proteins in ESCC patients which could predict the response to CRT.^[6]^ Among these proteins, p53 and p53R2 were independently correlated with chemoradiotherapy response or radio-sensitivity.^[6,7]^ Patients with negative expressions of p53 and p53R2 had 100% effective therapy response.^[7]^ Another gene, *PIK3CA*, also plays an essential role in ESCC and it would be a promising therapeutic target in the future. *PIK3CA* mutations in domains can also activate cell signaling pathway and promote ESCC cell growth. The invasion is via activation of the PI3K/AKT/mTOR pathway by the increase of the kinase activity of PI3K.^[8]^*PIK3CA* amplification could act as an independent poor prognostic factor in resected ESCC.^[9]^

Here, we report a case of an unresectable ESCC patient. Radiotherapy, chemotherapy and immunotherapy were not effective for this patient and he progressed very rapidly during CRT, accompanied by multiple organ metastases.

## 2 Case report

The patient was a 65-year-old man who presented with food obstruction, hoarse voice and choking on drinking water for 2 months, and pain behind the breastbone for 1 month. Gastroscopy revealed squamous cell carcinoma 23 to 25 cm from the incisors. Positron emission computed tomography examination showed that the middle and upper part of the esophagus was occupied, accompanied by multiple lymph node metastases in the right neck root and mediastinum. FDG uptake of the right lobe nodules of the liver was increased. Therefore, metastasis was considered. According to the eighth edition classification of UICC-TNM, the clinical stage of this patient was cT_4b_N_1_M_1_ (stage IVB). The patient had no indications for surgery in stage IVB and required local radiotherapy to relieve the current obstructive symptoms, hence CRT was planned. A single dose of 2Gy of radiotherapy was performed 5 times per week and the chemotherapy regimen was based on platinum drugs. Scored Patient-Generated Subjective Global Assessment score was 9. The nutrition group formulated a nutritional support treatment plan. According to the follow-up, stereotactic body radiotherapy treatment was considered optional for liver lesions. After obtaining the consent of patient and his family, the treatment was started. During the treatment process, symptoms such as low back pain had been occurred. Meanwhile, the liver lesions were larger than before in the liver magnetic resonance imaging (MRI) images, and lumbar MRI and bone ECT revealed new multiple bone metastases. Head MRI was normal without lesions. The lesions at the radiotherapy site (right neck root, mediastinal lesions) were absorbed and the range was reduced. All these results were observed in 1 week from the start of the first concurrent chemoradiotherapy. After discussing and evaluating within the department, the progression of the disease was considered. After communicating with the patient and family members, it was decided to add immunotherapy. Although the patient has not been tested for immunotherapy-related indicators, immunotherapy could be used as part of comprehensive treatment for patients with stage IV esophageal cancer. After obtaining the consent of the patient and family members, he was given camrelizumab 200 mg for one cycle. After 5 days, the patient began to develop cough, sputum, and bloodshot sputum. Chest computed tomography showed multiple lung nodules and metastasis was considered. The patient and his family gave up treatment, and his condition deteriorated rapidly after discharge. It took only 3 months from the onset of symptoms to death. In order to further find the cause of the patient's rapid progress, we performed genetic testing with his blood samples through high throughput DNA sequencing methodology (next generation sequencing; NGS) and found some meaningful somatic gene mutations as shown in Table 1.

**Table 1:** The results of whole-exome sequencing in the blood.

Gene	Exon	Nucleotide change	Amino acids change	Mutation rate (%)
TP53	exon10	c.1010G > T	p.R337L	17.81%
TP53	exon6	c.570_586delTCCTCAGCATCTTATCC	p.P191fs	15.71%
*PIK3CA*	exon21	c.3140A > G	p.H1047R	14.19%

## 3 Discussion

The TP53 gene is a tumor-suppressor gene coding for a multifunctional DNA-binding protein, that is, DNA repair, cell cycle arrest, apoptosis, and differentiation.^[10]^ More than 50% of human cancers and germline DNA with inherited cancer syndromes of the family could coexist with mutated TP53 gene.^[10]^ The mutant TP53 gene has been widely investigated in ESCC patients.^[11,12]^ Previous reports have suggested that mutated genes including TP53 (78% of patients), NOTCH1 (32%), ARID1A (13%), FAT1 (13%), CDKN2A (13%), and YAP1 amplification have been shown to be potentially useful biomarkers that can be used to predict treatment outcomes and identify patients with high risk of relapse.^[11,13,14]^ Chen et al reinforced the idea that TP53 mutation played an essential role in the initiation of the tumorigenesis of ESCC.^[14]^ Kang et al made a conclusion that mutant TP53 G245C and R273H could have lost TP53 function in carcinogenesis. It might play a role in clinical diagnosis and therapy of ESCC through generating more aggressive phenotypes and promoting cancer cell malignancy.^[12]^ Exon sequencing of blood samples from this patient showed that the 1010th nucleotide G located in exon 10 was replaced by nucleotide T, resulting in the 337th nucleotide in the corresponding protein sequence arginine (R) replaced by leucine (L), with a mutation rate of 17.81% in the sample. P53 R337L resulted in decreased TP53 tetramerization and trans-activation activity in cell cultures, which was expected to lead to a loss of function of the TP53 protein and inadequate chemoradiotherapy response.^[7,15]^ Similarly, a transcoding mutation in exon 6 in the sample with a mutation rate of 15.71% also resulted in loss of p53 protein function and promoted cancer cell proliferation, survival, and metastasis.^[16]^ A study showed that 36 patients with primary operable esophageal cancer were administered neoadjuvant therapy with cisplatin and fluorouracil (50% of tumors).^[17]^ The 2-year overall survival rates of patients with wild-type TP53 and mutant TP53 were 55.6% vs 16.7%, and the median tumor-associated survival was 34.2 vs 8.9 months, respectively. Besides, patients with mutant TP53 mutations have a three-fold increased risk of death (HR 3.01, CI 1.359–6.86).^[17]^

The *PIK3CA* gene, a gene in the PI3K/AKT/mTOR pathway which encodes the protein phosphatidylinositol 3-kinase (PI3K) and located on 3q26.3, can regulate multiple cellular events, including proliferation, cell motility, cell growth, apoptosis, and cell survival.^[18,19]^*PIK3CA* mutated was found in 7.4% of Chinese ESCC patients, and *PIK3CA* mutations independently correlated with worse overall survival in ESCC patients with age <59-year-old or family history of cancer.^[20]^*PIK3CA* overexpression independently correlated with local recurrence, and the female ESCC patients with *PIK3CA* overexpression had shorter overall survival.^[20]^ In addition, *PIK3CA* methylation rate in esophageal cancer significantly correlated with higher TNM staging.^[21]^

Exon sequencing of blood samples from this patient showed that nucleotide A at the 3140th position in exon 21 of *PIK3CA* was replaced by nucleotide G, resulting in the histidine (H) in the 1047th position in the corresponding protein sequence replaced by arginine (R), with a mutation rate of 14.19% in the sample. Accumulating evidence suggested that patients with *PIK3CA*-mutated ESCC might benefit from treatment with PI3K inhibitors, like PI3K inhibitor CYH33, which could play potent therapeutic activity against ESCC and could be used as a sensitizer during radiotherapy.^[22,23]^

In the present report, rapidly progressive esophageal cancer was observed in this patient. Even under active comprehensive treatment, it took only 3 months from the onset of symptoms to death. In the patient's blood samples, we found the R337L mutation in exon 10 of the TP53 gene and the P191fs mutation in exon 6. These mutations may lead to the loss of the TP53 protein function and prevent it from exerting the tumor suppressing effect. Instead, the mutant gene may be involved in the tumor promotion and progression.^[14,16]^ In addition, the H1047R mutation in exon 21 of *PIK3CA* was found, which may lead to the continuous activation of PI3K/AKT signaling pathway, thereby enhancing the growth, proliferation, and migration capabilities of tumor cells.^[8,24,25]^ Chen et al showed that co-mutation of TP53 and *PIK3CA* could be associated with poor survival in postneoadjuvant chemotherapy breast cancer patients and used as a potential prognosis marker in breast cancer.^[26]^ Although the role of TP53 gene and *PIK3CA* gene in the progression, treatment and sensitivity of esophageal cancer has been studied, the mechanism of their simultaneous appearance has not been demonstrated in relevant studies. We speculate that the reason for the rapid progression in this patient during active treatment might be related to this. The underlying mechanism and our hypothesis require further investigation.

Since the patient progressed rapidly during the treatment period, the camrelizumab was added to the treatment but the effect was not good. The genetic test results showed that the tumor mutation burden (TMB) was 19.333 Muts/Mb, which indicated a better immunotherapy effect. The reason for the poor effect in this patient may be related to the short evaluation time after medication.

## Acknowledgments

We acknowledge the patient and his families.

## Author contributions

**Conceptualization:** Ni Sun.

**Data curation:** Shuang Zhao, Ni Sun, Zetian Shen.

**Funding acquisition:** Xixu Zhu, Jing Li.

**Investigation:** Ni Sun, Xi Yuan.

**Validation:** Xi Yuan.

**Writing – original draft:** Shuang Zhao.

**Writing – review & editing:** Jing Li.
